# Serotonin syndrome due to concomitant use of linezolid and methadone

**DOI:** 10.1002/ccr3.6341

**Published:** 2022-11-05

**Authors:** Farnoosh Masbough, Soheil Roshanzamiri, Mitra Rahimi, Zahra Sahraei, Peyman Erfan Talab Evini

**Affiliations:** ^1^ Department of Clinical Pharmacy, School of Pharmacy Shahid Beheshti University of Medical Sciences Tehran Iran; ^2^ Department of Clinical Toxicology, Toxicology Research Center, Excellence Center of Clinical Toxicology, Loghman Hakim Hospital Shahid Beheshti University of Medical Sciences Tehran Iran; ^3^ Loghman Hakim Hospital Shahid Beheshti University of Medical Sciences Tehran Iran

**Keywords:** drug interaction, linezolid, MAO inhibitors, methadone, serotonin syndrome

## Abstract

Serotonin syndrome is a potentially life‐threatening adverse drug reaction typically caused by a single or combination of two or more medications with serotonergic properties due to increased serotonin release. Our case is a 60‐year‐old drug‐addict man who was admitted to the poisoning department of Loghman hospital with methadone poisoning. On the fifth day of hospitalization and after initiating the linezolid treatment for VAP, the patient began to run a fever with agitation, tremor, spontaneous clonus movement in the hands, and tachycardia. Due to patients' manifestations and after ruling out other diagnoses, serotonin syndrome was confirmed with the possibility of concomitant use of linezolid and methadone. Linezolid administration was promptly discontinued, and vancomycin therapy was initiated (1000 mg twice a day intravenously). Supportive therapies were performed. Finally, tremor, rigidity, and clonus movement disappeared within 48 h.

## Abstract 2

Before starting any new medication, any medications the patient are already taking should be investigated for interactions. Serotonin syndrome can be life‐threatening and could have been avoided if this had been done.

## 1 INTRODUCTION

Serotonin syndrome (SS) is a rare and potentially fatal condition first reported by Mitchell, which is caused by excessive stimulation of serotonin receptors in the nervous system.^1^ Serotonin regulates the central nervous system's attention, behaviour, and temperature (CNS). Serotonin syndrome has been linked to stimulation of the postsynaptic 5‐HT1A and 5‐HT2A receptors, though no one receptor is to blame. Any medication combination that increases serotonergic neurotransmission can cause serotonin syndrome. The condition can appear after initiating a single serotonergic medication or increasing the dose of serotonergic drugs in individuals sensitive to serotonin.^2^ Serotonin syndrome episodes involving a monoamine oxidase inhibitor are more severe and more likely to result in negative consequences, such as death. Features of this syndrome include mental status alteration such as delirium, anxiety and confusion, autonomic stimulation, for example, hyperthermia, tachycardia, tremor, and neuromuscular abnormalities such as myoclonus and rigidity.^3^ The leading cause of serotonin syndrome is drugs that elevate the level of serotonin in the CNS by affecting serotonin metabolism or acting as serotonin‐receptor agonists.^4^ The primary mechanism of its creation is as follows: inhibition of serotonin uptake, decreased serotonin metabolism, increased serotonin synthesis, increased serotonin release, and activation of serotonergic receptors. Serotonin syndrome can develop less than 24 h post‐ingestion of certain medications.^2^, ^4^

Linezolid is an oxazolidinone antibiotic against methicillin resistant *Staphylococcus aureus* (MRSA), Vancomycin‐resistant Enterococcus (VRE), and drug‐resistant *Streptococcus pneumoniae* (DRSP) infections. The structure of linezolid is similar to a selective, reversible monoamine oxidase inhibitor (MAO‐A) toloxatone used for depression treatment.^5^

Drugs, including SNRIs, TCAs, SSRIs, stimulants, and opioid analgesics such as tramadol, meperidine, methadone, and dextromethorphan, elevate serotonin levels and interact with linezolid.^3^, ^6^ Using linezolid with these drugs raises the serotonin concentrations in the central nervous system, resulting in serotonin syndrome.^5^ Food and Drug Administration warned against concomitant linezolid with serotonergic drugs and recommended a 2‐week washout in patients who previously received these medications.

In searches, we found only one case report describing serotonin syndrome with linezolid and methadone.^7^ We describe a case of SS in a 60‐year‐old drug‐addict man who was admitted to the emergency department.

## 2 CASE REPORT

A 60‐year‐old drug‐addict man was admitted to the poisoning department of Loghman hospital with dizziness, nausea, and vomiting. On examination, his blood pressure was 95/61 mmHg, heart rate 110 bpm, respiratory rate seven breaths per minute, and meiotic pupils with a negative neurological examination. The patient's medical history showed bipolar disorder and mental disability. In his past medication history, he used lithium (300 mg once daily), clonazepam (2 mg at bedtime), valproate sodium (500 mg twice a day), perphenazine (8 mg at bedtime), and methadone (unknown dose).

On admission, the patient was unconscious and did not respond to painful stimulation (Glasgow coma scale: 7). Laboratory findings included white blood cell count 14.2 × 10^9^/L, platelet 183 × 10^9^/L, creatinine 6.8 mg/dl, potassium 7.3 mg/dl, CPK 4218 U/L, and lactic acidosis. Drug concentration in the serum included lithium 2.6 mEq/L (therapeutic level: 0.6–1.2 mEq/L) and valproate sodium 217.6 μg/ml (therapeutic level: 50–120 μg/ml), with toxicology urine test being positive for methadone and benzodiazepine and negative for tramadol and cannabinoids. Except for methadone, other patients' drugs were placed on hold. A jugular catheter was inserted, and the patient underwent hemodialysis for 3.5 h in the emergency department. One dialysis session was sufficient, the patient's electrolyte disturbance resolved, and creatinine reached 3. He scored 10 on the Glasgow Coma Scale. After which, he was intubated and transferred to the intensive care unit.

On the third day of hospitalization, the patient was febrile (38.8°C), and the chest X‐ray revealed bilateral opacities, while computed tomography displayed consolidation and ground‐glass opacities (posterior segment of the upper lobes) in both pulmonary fields. So empirical antibiotics for aspiration pneumonia (ceftriaxone 1000 mg twice a day intravenously and clindamycin 600 mg three times a day intravenously) were initiated immediately. Blood and urine culture after 3 days was negative, but sputum culture was positive for *Staphylococcus aureus* (10^5^ CFU/cell). In the antibiogram, the microorganism was resistant to clindamycin and trimethoprim‐sulfamethoxazole. Based on sputum culture and resistance pattern, the antibiotics were changed to linezolid 600 mg twice a day intravenously. However, 2 days after the initiation of linezolid, the patient began to run a fever (39°C) with agitation, tremor, spontaneous clonus movement in the hands, and tachycardia (pulse rate 115/min). With suspicion of sepsis, a complete workup was performed.

Chest X‐ray did not change from the earlier state, and urine analysis did not show any abnormality. In addition, there was no evidence of seizures on the electroencephalogram (EEG), and brain CT scan findings were normal. Due to these manifestations, the first diagnosis for the patient was serotonin syndrome based on Hunter's criteria (sensitive and specific criteria for diagnosis of serotonin toxicity).^8^ Hunter's diagnostic criteria include at least one of the following features: spontaneous clonus, inducible clonus with agitation or diaphoresis, ocular clonus with agitation or diaphoresis, tremor and hyperreflexia, or hypertonia, temperature above 100.4°F (38°C), and ocular or inducible clonus. There is no particular laboratory test for the diagnosis of serotonin syndrome, but in some literature, an elevation of the total creatine kinase and transaminase levels, as well as leukocytosis, have been reported.^8^, ^9^ In the treatment of SS, linezolid administration was promptly discontinued, and vancomycin therapy was initiated (1000 mg twice a day intravenously). Hydration with 3 liters of electrolytic solution every 24 h, metoclopramide 10 mg three times daily intravenously, cyproheptadine 4 mg three times daily through a nasogastric tube, and benzodiazepine for agitation were administered. The patient's clinical condition gradually improved.

Within 48 h, tremor, stiffness, and clonus movement subsided. Although the patient's clinical condition had improved, his degree of consciousness had only marginally improved.

The patient's hemodynamic condition was stabilized, and the course of antibiotic treatment ended.

After 3 days of discontinuation of antibiotics, the patient was extubated, the level of consciousness was increased, and she made eye and verbal communication. Finally, after 1 week, he was transferred to the ward under stable conditions.

## 3 DISCUSSION

Serotonin syndrome is a potentially life‐threatening adverse drug reaction typically caused by a single or combination of two or more medications with serotonergic properties due to increased serotonin release. There are reports of serotonin syndrome with concomitant MAOIs (including linezolid, furazolidone, and procarbazine) and serotonergic antidepressants or drugs with SSRI‐like properties (meperidine, tramadol, methadone, dextrometophenyl, dextromethenepine).^10^, ^11^

In our case, replacement of linezolid (600 mg twice daily) with clindamycin based on an antibiogram for the treatment of pneumonia in a patient receiving methadone for addiction, temporarily led to a set of neurological features and mental state in the absence of other CNS pathologies leading to the diagnosis of SS.

Linezolid is an oxazolidinone antibiotic whose mechanism is through direct inhibition of protein synthesis by binding to the 23S fragment of the 50S ribosomal bacterial rRNA subunit. It has been reported to induce SS as interaction with almost any class of antidepressants.^6^

Baldo and Rose's study shows methadone is an intermediate risk factor for developing serotonin syndrome.^12^ In vitro studies have shown that methadone is more likely to inhibit serotonin reuptake than other opioids, leading to more serotonin syndrome among opioids.^13^ One case of SS was associated with methadone overdose and fentanyl and methadone use in burn injury.^14^

There is no specific evidence‐based treatment for the SS and most cases of this syndrome are mild and may be treated with the leftover of the offending agent and supportive care. However, critically ill patients may require neuromuscular paralysis, sedation, and intubation.^15^

## 4 CONCLUSION

The diagnosis of serotonin syndrome was difficult in the patient who presented with tremors, agitation, and clonus movement. Discontinuation of reference agents and treatment of symptoms is compelling. This syndrome must be prevented by educating patients to avoid self‐medication and limiting drug combinations.

## AUTHOR CONTRIBUTIONS

Farnoosh Masbough and Soheil Roshanzamiri participated in manuscript preparation; Peyman Erfan Talab participated in design of concepts, Mitra Rahimi and Zahra Sahraei participated in manuscript editing and review; all authors read and approved the final manuscript.

## CONFLICT OF INTEREST

The authors declared that there is no conflict of interest.

## CONSENT

Written informed consent was obtained from the patient to publish this report in accordance with the journal's patient consent policy.

## Data Availability

The data that support the findings of this study are available from the corresponding author upon reasonable request.

## References

[1] Mitchell RS . Fatal toxic encephalitis occurring during iproniazid therapy in pulmonary tuberculosis. Ann Intern Med. 1955;42(2):417‐424. 14350468 10.7326/0003-4819-42-2-417

[2] Volpi‐Abadie J , Kaye AM , Kaye AD . Serotonin syndrome. Ochsner J. 2013;13(4):533‐540. 24358002 PMC3865832

[3] Baldo BA . Opioid analgesic drugs and serotonin toxicity (syndrome): mechanisms, animal models, and links to clinical effects. Arch Toxicol. 2018;92(8):2457‐2473. 29916050 10.1007/s00204-018-2244-6

[4] Bartlett D . Drug‐induced serotonin syndrome. Crit Care Nurse. 2017;37(1):49‐54. 10.4037/ccn2017169 28148614

[5] Lawrence KR , Adra M , Gillman PK . Serotonin toxicity associated with the use of linezolid: a review of postmarketing data. Clin Infect Dis. 2006;42(11):1578‐1583. 16652315 10.1086/503839

[6] Quinn DK , Stern TA . Linezolid and serotonin syndrome. Prim Care Companion J Clin Psychiatry. 2009;11(6):353‐356. 20098528 10.4088/PCC.09r00853 PMC2805572

[7] Mastroianni A , Ravaglia G . Serotonin syndrome due to co‐administration of linezolid and methadone. Infez Med. 2017;25(3):263‐266. 28956544

[8] Mills KC . Serotonin syndrome. A clinical update. Crit Care Clin. 1997;13(4):763‐783. 9330840 10.1016/s0749-0704(05)70368-7

[9] Mason PJ , Morris VA , Balcezak TJ . Serotonin syndrome. Presentation of 2 cases and review of the literature. Medicine. 2000;79(4):201‐209. 10941349 10.1097/00005792-200007000-00001

[10] Dvir Y , Smallwood P . Serotonin syndrome: a complex but easily avoidable condition. Gen Hosp Psychiatry. 2008;30(3):284‐287. 18433663 10.1016/j.genhosppsych.2007.09.007

[11] Kulkarni RR , Kulkarni PR . Linezolid‐induced near‐fatal serotonin syndrome during escitalopram therapy: case report and review of literature. Indian J Psychol Med. 2013;35(4):413‐416. 24379509 10.4103/0253-7176.122245 PMC3868100

[12] Baldo BA , Rose MA . The anaesthetist, opioid analgesic drugs, and serotonin toxicity: a mechanistic and clinical review. Br J Anaesth. 2020;124(1):44‐62. 31653394 10.1016/j.bja.2019.08.010

[13] Gillman PK . Monoamine oxidase inhibitors, opioid analgesics and serotonin toxicity. Br J Anaesth. 2005;95(4):434‐441. 16051647 10.1093/bja/aei210

[14] Hillman AD , Witenko CJ , Sultan SM , Gala G . Serotonin syndrome caused by fentanyl and methadone in a burn injury. Pharmacotherapy. 2015;35(1):112‐117. 25615513 10.1002/phar.1528

[15] Ables AZ , Nagubilli R . Prevention, recognition, and management of serotonin syndrome. Am Fam Physician. 2010;81(9):1139‐1142. 20433130

